# An Inquiry into the Principles and Practice of Medicine, Founded on Original Physiological Investigations

**Published:** 1834-07-01

**Authors:** 


*An Inquiry into the Principles and Practice of Medicine, founded on original Physiological Investigations.* By G. CALVERT HOLLAND, M.D., &c. Vol. I.—London, 1834. 8vo. pp. 540.

No study is better fitted to teach men humility than that of medicine: affording unbounded scope for observation and for thought, presenting an object to which scarce any variety of knowledge is inapplicable, and opening a field in which every department of philosophy is cultivated with a common aim, it has been in all ages a favorite pursuit of the most gifted minds; yet to this hour it remains the most imperfect and unsatisfactory of the sciences. The seductive character of the pursuit, not less than its difficulty, has tended to retard its progress: and it has too often happened that active and ingenious minds, directed to a subject as delightful as it is interminable, have lost all severity of tone, and revelled instead of laboured. The "great principle of induction" has been talked of till it has become a cant, but it has been very little acted upon; and till facts, not the *inferences* which frequently go by that name, but plain unvarnished facts, universally admitted as such by all reasonable men, become the sole basis of medical doctrine, the science must remain conjectural, and the art empirical. It will no doubt be mortifying to see the limits of our science apparently contracted, instead of being enlarged; to see by far the greater portion of a vast fabric of thought melt into air, and to find ourselves in possession of only a small number of truths, as the mere foundation of a structure of much tardier growth; yet for this we must be prepared, if medicine is ever to approximate to the exact sciences. Many, we are aware, will maintain, that it is altogether incapable of such approximation, but this we do not admit: our knowledge will most likely always remain extremely circumscribed, but it may be exact as far as it goes; if it be otherwise, it is not knowledge at all. At present, indeed, medicine must be allowed to be the most conjectural of the sciences: but what has made it so? the habit of deluding ourselves with dreams of knowledge which we do not possess; the eagerness to generalise before a sufficient number of facts have been accumulated; impatience of the way, in short, and weariness of the labour. It is the desire to get on too fast that has made medicine a vague and unsatisfactory pursuit, and if we attempt, as we daily do, to arrive at truth in this science by roads which were never known to lead to it in any other, need we wonder at having to retrace our steps?

Medicine, as M. Magendie justly observes, is the physiology of the sick man; its basis therefore is the investigation of the healthy functions of the animal frame. This principle is not denied: but how much is it perverted in its application, and how many pathological theories are confidently reared on the most gratuitous suppositions as to the functions of organs or systems? It is needless to cite examples of this: the whole history of medicine is one great example.

Although the dependence of medicine on physiology is universally admitted, they have hitherto been little cultivated in any sort of philosophical connexion with each other: nor is this surprising, when we consider the extreme difficulty of placing their relations in a just light. With diffidence in entering on so arduous a subject, we subjoin a few remarks on some of the points which seem to bear most immediately and usefully upon it, premising that it is not our object to advance doctrines, but merely to throw out suggestions as a clue to experiment and observation,—the only true foundation for doctrine in this or any other science.

We may observe, in passing, that if those whose fertility of mind enables them to form new and ingenious conceptions would merely advance them as subjects of inquiry, instead of defending them as *theses*, and weaving them into baseless theories, they would confer a real benefit on science. All experiment implies some preliminary notion, some doubt to be resolved, or question to be decided ; something, in short, to determine the course of investigation in one channel rather than another. Hypothesis in itself is good, and without it experiment could have no beginning : it is only when it usurps the place of fact and observation that it becomes an evil.

*The Relation of the Nervous to the Vascular System.* The obscurity of the causes which influence the action of the blood-vessels, and more especially of the capillaries, constitutes a difficulty which everywhere confronts us in the application of physiology to medicine. In all our reasonings, whether on general or local disease, it opposes an insurmountable barrier to our further progress. To this question, therefore, the attention of the physiologist should be assiduously directed ; and we cannot help thinking that he might still derive very important aid from the researches of minute anatomy.

Daily observation demonstrates that the circulating system is strongly under the influence of causes which cannot be conceived to be immediately applied to it. The pulsations of the heart answer to a thousand impulses which can only be indirectly communicated ; and the arteries, to their minutest ramifications, have their action increased, diminished, or altered, by causes equally remote, and which evidently do not reside in the mechanism of the vascular system, or the influence of the fluid which it circulates.

Our attention is therefore naturally directed to the nerves, as the great medium of sympathy, and the most obvious chain of communication between distant organs. Now, it is certain that the action of the blood-vessels cannot be materially under the control of the nerves of sensation and voluntary motion, because that of the heart continues after the removal of the brain and spinal marrow ;* and that of the vessels of a limb remains unimpaired when both sensation and motion are

* Wilson Philip's Experimental Inquiry, p. 62.

destroyed by paralysis, or the division of the nerves. Again, it has been asserted, on good authority, that the fibres of the ganglionic nerves may actually be traced in the course of the principal arteries, till they become so minute as to elude the eye, affording every presumption that they still accompany the vessels to their minutest ramifications.* Supposing these observations accurate, how does the question stand? We have an organ whose function is to be assigned, and a function whose organ is to be discovered: we are all but certain, on the one hand, that local changes in the action of the minuter vessels do not arise from the mechanism of the vascular system itself, or the influence of the contained fluid on the vessels. On the other hand, if the use of the fibres of the sympathetic, interwoven with the coats of the arteries, be not to influence and regulate their actions, the utmost ingenuity cannot devise even a probable function for them. We ought therefore, by a further appeal to minute anatomy, to endeavour to ascertain whether the fibres of the sympathetic can actually be traced to the situation in which they are alleged to have been seen: if they can, there seems to be no reason why we should not adopt, with some confidence, an opinion which is corroborated by many striking phenomena of the living system, contradicted, as far as we know, by none, and whose evidence, though short of demonstration, would afford that strong degree of probability on which we are contented to reason in many other matters of at least equal importance.

*Animal Temperature.* This subject is obviously one of great moment in its relations to pathology. The two leading theories, that which connects the temperature of the body with respiration, and that which attributes it to the influence of the brain, have both striking, though not conclusive experiments in their favour; and the former is supported by an extensive and beautiful chain of analogies; yet we have little hesitation in stating our belief, that they ought both to be relinquished, because they are both manifestly incompatible with the facts of disease. Confront the chemical theory with the following cases related by John Hunter.

"Case I. A gentleman lying apoplectic, whilst in bed, would become suddenly cold, and as quickly would become extremely hot; and here there was no alteration in the pulse or breathing.

"Case II. A man lying comatose in consequence of injury to the brain, and breathing with only half his usual frequency, was yet extremely hot.

"Case III. A boy, who appeared to be dying, breathed so slow

* Vide, in particular, Copland, Notes to Richerand, p. 556.

that twenty-three seconds elapsed between each act of breathing: the pulse was weak and very slow; the body of purple hue, the blood not passing often enough through the lungs to acquire its scarlet hue: he was very warm, though in the midst of winter, and in a room without a fire."—(*Parkinson's Hunterian Reminiscences*, p. 14.)

These cases are not solitary; instances equally subversive of Crawford's theory occur continually in the course of ordinary practice, rapid alternations of temperature being observed without any change in the respiration at all commensurate with the effect produced; and where such effect cannot be connected, with any reasonable probability, with the state of cutaneous evaporation which is supposed to modify the influence of the other chemical causes.

Brodie's theory is still more obviously at variance with pathological and other facts: thus, in cases of injury of the vertebræ, where all the parts below the seat of the injury are entirely deprived of sense and motion by the interception of their communication with the brain, the temperature of these parts often remains perfectly natural. Again, children have been born without any brain, who nevertheless evinced no defect of animal temperature. Nor has the spinal cord any better claim to be considered as the source of vital heat, since complete paralysis of the nerves derived from it is frequently unattended with any diminution of temperature in the limb to which they are distributed.

The hypothesis of Sir E. Home, which refers the generation of animal heat to the ganglionic system, is worthy of considerable attention, though very feebly supported and reasoned on by its author. (*Quarterly Journal of Science*, vol. xx.)

The opinion of John Hunter, as far as it can be collected from his writings, was, that living parts had an inherent power of regulating their own temperature according to their necessities; an opinion which may be considered by some as a sort of begging of the question, but which has some very striking facts in its favour, and is perhaps, on the whole, less at variance with the results of observation than any which has succeeded it.

The subject of animal temperature is evidently one on which we are in want of more facts; these the patient researches of the physiologist may possibly supply, while the phenomena of disease will afford a good touchstone on which to try the validity of the conclusions derived from them.

*General Anatomy.* Bichat's doctrine of the tissues has been hailed as the dawn of a new era in medicine; but, whatever its future utility may be, it seems to us that the influence it has hitherto exerted on that science has been greatly overrated; so far, indeed, from forming the basis of any very important practical views, it has given birth to little else than generalizations whose accuracy is extremely doubtful. The phrase "properties of tissue" has got into current use, without perhaps any very definite idea being attached to it; in virtue, however, of the said properties of tissue, a certain similarity has been assumed to exist between the morbid conditions of the same texture in whatsoever part of the body it may be found; but we believe it may be correctly affirmed, that any such analogy is hitherto known to hold good only when there is a perfect similarity in the functions to which the given texture ministers.

Take, for example, the serous membranes: it is true that there is a remarkable uniformity in the pathology of these membranes throughout the body; thus, inflammation presents in all the same phenomena, it has the same terminations, is attended by the same kind of constitutional symptoms, and is affected in the same manner by therapeutical agents; but it is also true, that there is an almost perfect similarity of distribution and function among these membranes; they all line shut cavities, and afford a superficial covering to important organs.

Synovial membranes, again, present a like uniformity in their morbid conditions; but here also we have the same resemblance of distribution and function. All these membranes line articular cavities, whose surface they lubricate by the secretion of a similar fluid.

Many other examples might be adduced, which all go to prove the same thing; hence all pathological reasoning founded on properties of tissue is premature, since the analogies observed are found to be universally connected with uniformity of function; and there is no proof that they would hold good independently of such uniformity: no proof, for example, that a serous membrane, if conceived to line an excretory canal instead of a shut cavity, would retain its pathological similarity to the other serous membranes.

Upon the whole, then, it appears that the only pathological inference at present deducible from the doctrine of the tissues is, that, when the healthy functions of parts are similar, the derangement of these functions in disease will also be similar; a conclusion so obvious, that we do not require the assistance of general anatomy to arrive at it; and, if this study has in any degree furthered the progress of medicine, it has been rather by affording a convenient mode of classifying morbid appearances, than by throwing any additional light on the nature or results of morbid actions.

It has moreover unfortunately happened, that the too hasty generalization of the doctrine of the tissues has led to positive errors in pathology and false principles in practice.

The origin of these errors may, indeed, be traced to Bichat himself, the author who first reduced the facts of general anatomy to a systematic form. There can be little doubt that this distinguished physiologist borrowed his first ideas on the subject without acknowledgment from Andrew Bonn's treatise, "De Continuationibus Membranarum."

Bonn's views were purely anatomical, and related only to the continuity of surface in different membranous textures throughout the body. Bichat, adopting his facts, and adding to their number, observed that similarity of property frequently accompanied the continuity of surface; and hence appears to have prematurely adopted the notion that such correspondence was universal: the result was the classification of certain continuous surfaces as homogeneous textures which have in fact no right to be so considered.

Subsequent authors have not been slow in extending this error, for an instance of which we need go no further than mucous membrane, a texture very generally diffused, and of great importance in its pathological relations. The several membranes classed together under this denomination are frequently considered by medical writers as constituting a tissue which is nearly the same throughout the body; and a sufficient similarity is assumed in their diseases to be made the basis of practical precepts generally applicable to them. Now, so far is this from being the fact, that scarcely any two mucous membranes agree precisely in their external appearance, in their functions, in the influence of physical agents upon them, or in the phenomena and results of their diseased actions. Their functions are most diversified: in the stomach we find mucous membrane ministering to the secretion of a fluid different from all others; in the small intestines it forms an absorbing surface for the purposes of nutrition; in the cells of the lungs it forms a permeable web, allowing the access of the air to the blood; in the urinary bladder it lines a reservoir for containing a highly irritating fluid; in the urethra it lines an excretory canal; in the nose, mouth, and ear, it affords a surface for the distribution of the nerves of sense; and in the conjunctiva of the eye it constitutes the outward protection of a very delicate organ. Even the common secretion from which the mucous membranes derive their name is not similar in any two of them: we have, indeed, as Dr. Craigie justly observes, certain *mucous* membranes which secrete no mucus at all,* and whose very name is consequently a solecism.

Physical agents affect different mucous membranes in different ways; cold and damp air very frequently inflames the membrane of the nose, but has no such effect on that of the mouth; urine produces no effect on the mucous surface of the bladder, but would severely irritate that of the eye.

The phenomena and terminations of diseased action are dissimilar in different membranes of this class: thus, inflammation of the membrane of the nose is not attended with pain, but with a disagreeable sensation of heat, and, if not resolved, terminates in increased secretion of mucus, or secretion of pus without solution of continuity; inflammation of the lining membrane of the mouth occasions considerable pain, and never terminates in a secretion either of mucus or pus, but frequently in ulceration.

Notwithstanding all these diversities, which plainly show that mucous membrane is not one tissue, but many tissues, we find certain general precepts confidently given for the treatment of its diseases: thus it is a current maxim with many practitioners, that inflammation of mucous membranes does not bear bleeding well. This may be true of some of them, but it is most untrue of others: common conjunctival ophthalmia, for instance, not only bears bleeding well, but requires a larger use of this remedy than inflammation of the same extent in any other membranous surface throughout the body.

We cannot pursue the details of this subject further, but we think that all attempts to adapt the doctrines of general anatomy to medicine had better be laid aside till their applicability is better established, and the mode of their application better understood. There is a subject which may be considered as a branch of general anatomy which has received little or no attention, but from which some interesting conclusions might possibly be derived. We allude to an investigation and comparison of the properties of organised textures which are produced by morbid actions.

Morbid growths, considered in the mass, have indeed been the objects of much minute description; but the different textures of which they are composed,—the relation of these textures to each other, and to the healthy tissues which they more or less resemble, have never yet been subjected to scientific analysis. Morbid anatomy, in one sense of the term, is cultivated with sufficient diligence, but it is chiefly conversant with the *products* of destructive processes,—with the *debris* of

* Elements of General and Pathological Anatomy, p. 660.

the fabric, rather than the causes which led to its dilapidation; and hence its use is rather to localize disease than to explain it. But disease creates as well as destroys, and a careful comparison of the various forms of morbid organization, (in other words, a general anatomy of disease,) might perhaps be brought to bear advantageously both on physiology and pathology. To give an example of our meaning: a sinus is lined by a sort of membrane bearing more resemblance to mucous membrane than to anything else; the cavity of an abscess has a somewhat similar lining. Wherein do these membranes differ from healthy mucous membranes, or from each other? Has that lining the sinus any more resemblance to the membrane of an excretory canal than that lining the abscess? Questions of this kind are always worth proposing: it is true they may generally lead to nothing, but sometimes they may afford the clue to important investigations.

Having alluded to morbid anatomy, we may perhaps be indulged in a momentary digression on this subject. Important as the study is in itself, and indefatigably as it is followed up, we think it extremely doubtful whether its operation at the present moment is to advance or retard the progress of medicine. If it were philosophically pursued, there could be no such doubt; but it is too often most unphilosophically pursued. A great number of pathologists have fallen into an error as pernicious as it is unaccountable—the disposition to couple every disease with some change of structure; which will, in reality, be found to amount to nothing more nor less than the identification of all actions with those of the vascular system. The pathologists we speak of are aware of the fact that every organ is originally constructed by vessels; that its waste is occasioned and repaired by their action; that its form, consistence, colour, all its properties manifest to the eye or touch, depend upon such action: accordingly, when they find an organ changed in any of these properties, they justly refer the change to disordered action of the vessels; and when disordered vascular action has subsisted for a certain length of time, they rationally expect an obvious change in the structure of the organ. Now, if they would be content to trace and record the ravages of different degrees and kinds of disordered vascular action, wherever they are actually to be found,—in short, if they would describe what they see, and no more, they would be philosophically and usefully employed. But they are not content with this: they insist on change of structure as the necessary consequence of diseased actions, in which there is no reason to believe that vessels are immediately concerned. Thus, where there has been derangement of the intellectual functions, they must needs have induration, or softening, or other apparent change, in the structure of the brain;—if the sensibility of an organ has been for some time morbidly increased, they hold it impossible but that its texture must be changed; forgetting that there is no reason to suppose that the action of the vessels has any immediate connexion with the functions in question, that vascular action may be one thing, and nervous action another. A disease, say they, which cannot be seen, is no disease at all; and, if they cannot see what they want by the ordinary exercise of vision, they avail themselves of the grandest of all microscopes, a favourite notion; and thus, while professing to accumulate facts, they are merely torturing nature into a conformity with preconceived views, and justifying the saying, that there are in medicine more false facts than false theories. In consequence of this stupid error, (for such we really must call it,) many industrious persons are expending years of labour in seeking for what they will never find, or imagining what they cannot see.

*The Origin of Diseases.* The animal frame is evidently destined to pass through the successive periods of growth, maturity, and decay, and its organization is prospectively adapted to this course; so that, setting aside the influence of extraneous causes, there would be nothing to occasion a deviation from this natural progress, and the individual would live free of disease, and die when his organism was worn out, as is frequently observed to be the case in savages, and occasionally in civilised men. In a healthy animal body, then, we must seek for the origin of disease in the influence of extraneous agents, and among these our chief attention is naturally directed to such as are known to be most constant and most influential in their operation. But, again, it is obvious that only certain parts of the frame are immediately exposed to the action of these causes: the surface of the alimentary canal is liable to be irritated by unwholesome ingesta; the external surface of the body is exposed to the continual vicissitudes of heat and cold, dryness and moisture, and acted on, more or less, by all the physical changes of the atmosphere; many other parts, however, are not so exposed; no external agent, for instance, is immediately applied to the substance of the heart, none to the texture of the peritoneum; yet these parts become the seat of a variety of diseased actions. Is it not probable, then, that the origin of a great number of diseases is to be sought for in sympathy; the morbid cause acting immediately on the part to which it is applied, and affecting remote parts which consent with it according to laws which are most likely invariable, though hitherto undetermined? What a vast variety of remote diseases are known to originate in the stomach and intestinal canal, and what an impulse has the knowledge of this fact given to the theory and practice of medicine! May there not be a number of analogous facts equally important and equally applicable; and may we not, at this moment, be erroneously referring many diseased actions to sympathy with the digestive organs, which are in reality to be attributed to sympathy with other organs not less exposed to noxious agents, and exerting no less powerful and extensive an influence on the system at large? A patient, for example, complains of general languor, debility, and depression; he is somewhat emaciated; his complexion is sallow, and his appearance haggard; and all this without any local pain, or febrile affection of the system. We might at first be inclined to refer his symptoms to derangement of the digestive organs; but what if the tongue be clean, the appetite sufficiently good, the bowels regular, and the alvine excretions natural? We must seek for some other cause, and we may perhaps find it in a chronic affection of the bronchial membrane, indicated by an increased secretion, causing a degree of mucous rattle; and somewhat impaired respiration, without any evidence of disease of the pulmonary tissue. We are not imagining a case, but describing one of frequent occurrence. Now that such affection often arises from the local action of atmospheric causes on the bronchial membrane, is rendered probable by the fact that many persons are almost constantly subject to it in some localities, and perfectly free from it in others, their diet, habits, and other circumstances, remaining the same.

Again, a disturbed state of the general functions of the system may often be apparently traced to a dry and imperspirable state of the skin, which may originate from causes acting locally upon it, since the symptoms are sometimes most effectually relieved by remedies acting directly on the surface, as tepid bathing and friction. In applying this view of the origin of diseases, we should attend to the various organs which, from their situation, or relations to external agents, are most liable to injurious impressions: it should also be kept in mind, that some symptoms may arise from the sympathies between the exposed organs and distant parts, and others from the derangement of important functions exercised by such organs. We may adduce the following examples.

1. The whole range of the alimentary canal may be irritated by noxious ingesta; and here we have to consider not only the sympathies of the several parts of this canal with distant organs, but the disturbance of the nutrient function which is essential to the whole system.

2. The respiratory passages may have their lining membrane affected by the qualities of the atmosphere; here we have to take into account not only the sympathies which may be exercised by these parts, but the insufficient accomplishment of those chemical changes in the blood which are necessary to the healthy functions of every organ in the body.

3. The surface of the body is exposed to the action of atmospheric and various other causes; and disease in distant parts may arise not only from sympathy with the cutaneous texture, but from the disturbance of its absorbent and exhalent functions, its influence on the capillary circulation; and, consequently, on the distribution of the blood.

4. The centres of the nervous system are continually impressed through the medium of the organs of sense, and the continuance of too intense excitement of any of these may thus become a source of disease; nay, it were easy to point out instances in which it manifestly is so.

We can by no means pretend to enumerate the various ways in which external agents may produce local impressions communicable by sympathy to distant organs; we merely propose the subject, which we think might yield some valuable results to patient and philosophical inquiry. The train of reasoning is exceedingly obvious, yet we are not aware that it has ever been followed up to any extent, or even that the conditions of the question have ever been distinctly stated.

In pursuing this subject, it would be necessary carefully to reconsider the various kinds of sympathy hitherto recognised by physiologists; to apply them where it is possible, thus extending the knowledge of their laws; and to ascertain whether there be any other kinds of sympathy which have as yet escaped observation.

Finally, it is to be remembered that, although many diseases may probably be traced to the physical causes above alluded to, there is little doubt that a host of others proceed from the operation of causes which are altogether hidden from us; which act on we know not what part, and may gain as easy access to one part as another; as contagions, of which we understand nothing but their effects, electricity, and many other things 'twixt heaven and earth, which are not dreamed of in our philosophy.

*The Influence of the Mind on the healthy and diseased*

*Functions of the Body.* This subject, though evidently one of vast extent and importance, has given rise to so little scientific investigation, that a man, in search of recorded facts connected with it, would perhaps find more to his purpose in the works of poets, painters, and sculptors, than in those of physiologists and physicians. The few works published on this subject, though creditable to their authors, and useful, as forming at least a commencement of inquiry, have been little attended to; and we believe we may affirm with truth, that the influence of the mind on the animal frame remains as an open and almost uncultivated tract, which may yield an ample recompense to those who have the boldness to enter, and the perseverance to explore it. That there are facts on this subject is obvious, that they may be capable of generalization, and afford useful inferences, is by no means impossible; at all events then it would be worth while to collect and record them.

We must however observe, that if this study is to be pursued with any prospect of success, the inquirer must divest his mind of a very gratuitous hypothesis, which has become so generally prevalent as to be regarded by many as a simple matter of fact; namely, that we are actually acquainted with the seat and organ of the mind. The ancients believed the heart to be the seat of the moral feelings, and the liver of the passions, conceits at which we now laugh; but, in assuming the brain to be the organ of the intellectual faculties, or that these faculties have any organ at all, we may, peradventnre, be indulging in a conceit altogether as airy. A mind of philosophical training will not easily admit the truth of a doctrine which involves an utter incongruity between the cause and the effect; which attributes to the brain, a *material* organ, the production of thought, an *immaterial* result. All material operations, which we know to be such, whether the matter engaged in them be inert or organised, produce a result some way or other relating to matter: mechanical operations produce motion, by which matter is made to change its position; chemical operations produce changes in the sensible properties of bodies; the operations of organised matter also produce material results; muscular action causes motion, which may be measured like any other kind of motion; the action of the liver produces bile, which is a fluid, and has chemical and mechanical properties like all other fluids. But what are the products of the mind's operations?—ideas, various modes of thought and feeling. Now we should be glad to know what properties these have in common with matter; what is the colour of a recollection; what the specific gravity of a doubt; what the chemical properties of a wish? It is evident that there is not a single property in which any one mental product resembles any one material product.

Surely, then, it is absurd to attribute to a material organ results which have nothing in common with matter;—as absurd to suppose that the brain can produce thought, as that we could produce a brain by thinking of one; the absurdity in either instance consisting in an entire incongruity and want of relation between the cause and effect. The absurdity is the same as if one were to talk of producing sounds by adding numbers together: the two things have nothing whatever to do with each other.

We admit, however, that all this is but matter of opinion; not hesitating, at the same time, to state our own, that the doctrine of the brain's thinking is flat nonsense. All we contend for is, that a mere assumption, resting on no kind of proof, should not be allowed to influence a science, whose only legitimate foundation is in observation and experiment.

There are various other physiological subjects which have very important relations to pathology, as the influence of the several periods of development in the aggravation or correction of pre-existent diseases, and the production of new ones; the effects of the periodical changes which are observed to take place in the animal economy; morbid diatheses; hereditary predispositions to disease; and many additional topics, on which it would be much easier to exhaust the patience of the reader than to say anything at all to the purpose. The consideration of the action of remedies also forms an important part of the application of physiology to medicine; but, as all that can be said on this subject is comparatively familiar, we shall not further protract this article, over which it is more than probable that the gentle reader has already fallen asleep.

